# Feasibility and acceptability of a brief routine weight management intervention for postnatal women embedded within the national child immunisation programme in primary care: randomised controlled cluster feasibility trial

**DOI:** 10.1186/s13063-020-04673-9

**Published:** 2020-09-01

**Authors:** A. J. Daley, K. Jolly, H. Bensoussane, N. Ives, S. A. Jebb, S. Tearne, S. M. Greenfield, L. Yardley, P. Little, N. Tyldesley-Marshall, R. V. Pritchett, E. Frew, H. M. Parretti

**Affiliations:** 1 School of Sport, Exercise and Health Sciences, Loughborough University, Loughborough, Leicestershire LE11 3TU UK; 2 Institute of Applied Health Research, University of Birmingham, Edgbaston, Birmingham, B15 2TT UK; 3 Birmingham Clinical Trials Unit, Institute of Applied Health Research, University of Birmingham, Edgbaston, Birmingham, B15 2TT UK; 4 Nuffield Department of Primary Care Health Sciences, University of Oxford, Oxford, OX2 6GG UK; 5 School of Psychological Science, University of Bristol, Bristol, BS8 1TH UK; 6 Department of Psychology, University of Southampton, Southampton, S017 1BJ UK; 7 Faculty of Medicine, University of Southampton, Southampton, SO17 1BJ UK; 8 Norwich Medical School, University of East Anglia, Norwich, Norfolk NR4 7TJ UK

**Keywords:** Weight, Postnatal, Diet, Immunisations, Nurses, Primary care, Randomised feasibility trial

## Abstract

**Background:**

The prevalence of obesity in women continues to rise and pregnancy is a high-risk time for excessive weight gain. The period after childbirth represents an opportunity to offer women support to manage their weight. The primary aim here was to investigate the acceptability and feasibility of delivering a self-management intervention to postnatal women to support weight loss, embedded within the national child immunisation programme.

**Methods:**

The research involved a randomised controlled cluster feasibility trial. Data were collected at baseline and 3 months later. Twenty-eight postnatal women living with overweight or obesity were recruited via Birmingham Women Hospital or general practices. Babies are routinely immunised at 2, 3 and 4 months of age; the intervention was embedded within these appointments. The intervention involved brief motivation/support by practice nurses to encourage participants to make healthier lifestyle choices through self-monitoring of weight and signposting to an online weight management programme, when they attended their practice to have their child immunised. The role of the nurse was to provide external accountability for weight loss. Participants were asked to weigh themselves weekly and record this on a record card or using the online programme. The weight goal was for participants to lose 0.5 to 1 kg per week. Usual care received a healthy lifestyle leaflet. The primary outcome was the feasibility of a phase III trial to test the subsequent effectiveness of the intervention, as assessed against three stop-go traffic light criteria (recruitment, adherence to regular self-weighing and registration with an online weight management programme).

**Results:**

The traffic light stop-go criteria results were red for recruitment (28/80, 35% of target), amber for registration with the online weight loss programme (9/16, 56%) and green for adherence to weekly self-weighing (10/16, 63%). Nurses delivered the intervention with high fidelity.

**Discussion:**

Whilst participants and nurses followed the trial protocol well and adherence to self-weighing was acceptable, recruitment was challenging and there is scope to improve engagement with the online weight management programme component of the intervention.

**Trial registration:**

ISRCTN 12209332. Registration date is 04/12/18.

## Background

Obesity is known to have negative effects on the physical, mental and social health of the population [1]. Women’s main child-bearing years (around 25–34 years) hold the highest risk of weight gain compared with men or women of other age groups [2]. Irrespective of pre-pregnancy weight status, most women gain above the recommended weight during pregnancy [3, 4]. It is estimated that a woman with a body mass index (BMI) of 29.4 kg/m^2^ will on average gain about 14–15 kg during pregnancy and at 1 year after birth 5–9 kg is retained [5–8]. One explanation for excess weight gain during pregnancy is the traditional ideology of ‘eating for two’ [9], a common explanation given by women who feel pregnancy is a time in their lives where they can eat without restraint. In addition, physical activity typically decreases during pregnancy [10]. The fact that most women do not lose extra weight gained during pregnancy is important because postnatal weight retention contributes to the development of obesity in later life and increases the risk of complications in any future pregnancy [11, 12]. Research shows that postnatal women who are living with overweight would prefer to weigh less, are interested in implementing weight loss strategies and would welcome support to help this outcome, as little support is currently offered by the NHS [13]. In the absence of evidence to support the benefit of weight management interventions during pregnancy, postnatal interventions are increasingly important [14–16].

A systematic review of systematic reviews (of randomised controlled trials: RCTs) evaluated the effectiveness of weight management interventions in postnatal women [17]. Women randomised to a lifestyle intervention had significantly lower weight than comparators (mean difference of − 1.7 kg: 95% CI − 2.3 to − 1.1 kg) post intervention. However, many of the interventions tested were very intensive and tailored lifestyle-based programmes that were often delivered by skilled health professionals. Despite evidence suggesting that some of these interventions were effective, the NHS lacks the resources to scale-up these intensive interventions. More specifically, resource-intensive interventions cannot be delivered to all 820,000 women who give birth annually in the UK, 520,500 of whom will be living with overweight at the start of pregnancy [18]. Furthermore, the acceptability of some of the interventions evaluated in the review was low with high drop-out rates and/or poor levels of engagement. Most trials had recruited small sample sizes with short follow-up periods. Therefore, high-quality trials are required that test more acceptable and low-cost weight management interventions, designed to be suitable for all postnatal women who would like to lose weight after childbirth.

One approach that does not place additional burdens on the healthcare workforce is the provision of brief interventions embedded within existing health care consultations, in line with the ambition of the NHS to ‘Make Every Contact Count’ [19]. Current evidence suggests that brief interventions and/or interventions that encourage self-regulation for the treatment of overweight/obesity can be effective [20, 21], but reviews have not found any RCTs that have tested a weight management intervention embedded within routine health care appointments for postnatal women. The primary objectives of the study were to assess whether the trial appealing to women (via assessment of the recruitment rate to ensure a full phase III trial was feasible); whether the intervention was acceptable; whether the intervention had any adverse impact on infant immunisation rates; and the number of women who completed the trial and completed the trial questionnaires.

## Methods

### Design

A randomised controlled cluster feasibility trial design was used to assess the feasibility and acceptability of the intervention. GP practice was the unit of randomisation. Cluster randomisation helps to avoid the possibility of contamination occurring in usual care participants. In this trial, practice nurses were trained to deliver the intervention. If an individual randomisation design had been used, nurses could potentially use aspects of their training with women assigned to the usual care group. It was also possible that women registered at the same practice could potentially share information and intervention resources. To avoid the possibility of contamination, practices (clusters) were randomised to either the weight management intervention or comparator group. Favourable ethical approval for this study was obtained from NRES Black Country Ethics Committee (Reference Number: 236462). The full trial protocol has been published [22]. This study took place in Birmingham, UK.

## Methods of recruitment and initial screening

### Birmingham Women’s Hospital

Computerised systems at the Birmingham Women’s Hospital (BWH) allowed for systematic identification of all postnatal women who had recently given birth to reduce the potential for recruitment and selection bias. Every 2 weeks BWH conducted searches of women aged ≥18 years who had recently given birth and were registered at participating general practices. A trial invitation letter and the participant information sheet (PIS) were mailed to these women. Women did not receive their invitation letter until at least 4 weeks post-delivery. Women were linked to general practice by their medical records. BWH applied the following initial screening criteria before sending the invitation letters to women: confirmed the participant was aged ≥18 years, had given birth at least 4 weeks previously and was registered at one of the participating practices. Mothers whose babies had died or had been removed from their care at birth were excluded. The invitation letter included the research team telephone number which women could call if they were interested in participating, or they could post a reply form. Further screening by telephone was conducted by the research team prior to the baseline home visit to establish additional eligibility criteria; self-reported height and weight to check BMI ≥25 kg/m^2^; confirmation that participants were planning to have their child immunised and had not yet attended the first child immunisation appointment; were not already involved in a weight loss programme or a weight management trial; and participants were willing to give consent to notify their GP of their participation in the trial. Assessment of full eligibility was completed at the baseline home visit (see later).

### Direct recruitment through GP practices

Towards the end of the study recruitment period, recruitment via BWH was supplemented with recruitment strategies directly via practices. Posters were made available for viewing on GP practice waiting room television screens. Participants could be informed about the trial directly from baby check clinics, postnatal check-ups or at any other appointment with the GP. The researcher provided these potential participants with the letter of invitation and PIS. If women were interested, they were screened at the practice by the researcher or later telephoned to establish eligibility. If all initial screening eligibility criteria were met, a baseline appointment was made for a researcher to visit potentially eligible participants at home to fully confirm their eligibility.

### Establishing full eligibility at the baseline home visit and informed consent

Written informed consent was a two-stage process. At the baseline home visit, a researcher obtained written informed consent to collect further screening data to fully confirm eligibility. The researcher measured participants’ height and weight to objectively confirm the BMI eligibility criteria (≥25 kg/m^2^). The researcher also confirmed that participants had not been diagnosed with a serious mental health difficulty requiring hospitalisation or been diagnosed with anorexia and/or bulimia in the past 2 years. Participants who met all the eligibility criteria were asked to provide written informed consent to the trial. Baseline assessments were then undertaken.

### Randomisation

Practices (clusters) in Birmingham and Solihull were invited to participate. The practices were randomised in a 1:1 ratio to the weight management intervention or usual care using minimisation for practice list size (large: 6000 or more; small: under 6000 patients) and Index of Multiple Deprivation (IMD) score [23] to ensure balance in these factors across the two trial groups. The IMD was based on the postcode of the practice; IMD score ranges from 1 to 32,844 and was divided into tertiles of high, medium and low levels of deprivation. The Birmingham Clinical Trials Unit (BCTU) created a computer-generated randomisation list to allocate practices to the trial groups. The randomisation list was held securely by BCTU. Once all the necessary approvals were in place, practices were randomised centrally by the trial statistician, and those practices randomised to the intervention group received the required training to deliver the intervention prior to opening for the trial. To maintain allocation concealment of trial group at the start of the study, randomisation of the first practices occurred when three practices were ready to open (except for need for trial intervention training). Thereafter, practices could be randomised once they were ready to proceed.

### Masking

It was not possible to mask participants or those providing the intervention to group allocation. We do not believe that this would have introduced bias because the aim was to assess the feasibility of undertaking a large phase III cluster RCT and these outcomes are not affected by knowledge of group allocation and because the data relating to feasibility outcomes were not collected during the home visits.

### Intervention

#### Overview summary

The intervention was deliberately designed to be multi-component as evidence suggests that such interventions lead to more favourable weight outcomes during the postnatal period [24]. Babies are routinely immunised at 2, 3 and 4 months of age, which has a coverage rate of 94% in the UK; the intervention was embedded within these routine immunisation contacts, so no additional visits by participants were required [25]. The intervention involved nurses encouraging participants to make healthier lifestyle choices through self-monitoring their weight and signposting them to an online weight management programme (POWeR) for support [26]. Participants were asked to weigh themselves weekly and record this on a weight record card that was attached to the child health record ‘red book’ where infant immunisations are recorded or using the online programme. This allowed nurses to check that participants were weighing themselves regularly, whilst the POWeR programme provided personalised information based on weight gain/loss progress.

#### Weight loss goals

No clinical guidelines that specify rates of healthy weight loss for postnatal women are available, but for the adult general population, NICE recommend 0.5–1 kg per week [27]. Participants were therefore advised to aim for a weight loss goal until they had achieved a BMI ≤25 kg/m^2^ and were no heavier than their pre-pregnant weight.

#### External accountability

The role of the nurse was to provide encouragement, regular external accountability and to signpost participants to using POWeR for weight loss information. Nurses did not provide any counselling about diet/physical activity, they simply weighed participants at each immunisation and recorded this weight. Someone who is regularly weighed is more likely to maintain weight goals when they know their progress will be monitored by another individual [28, 29].

#### Online weight loss programme (POWeR: Positive Online Weight Reduction)

Nurses signposted participants to the POWeR online weight loss programme for weight loss support and assistance with goal setting, action planning and implementation of changes to their lifestyle [26]. POWeR is a self-guided online intervention to support weight management. Participants choose either a low energy eating plan (a reduction of around 600 calories a day) or a low carbohydrate eating plan. Users are also encouraged to increase their physical activity levels. POWeR focuses principally on fostering users’ self-regulation skills for autonomously self-managing their weight, rather than providing detailed dietetic advice. Users of the programme are taught active cognitive and behavioural self-regulation techniques to overcome problems such as low motivation, confidence or relapse. Information about breastfeeding and weight loss was added to the programme for this trial. Participants were encouraged to continue to use the website weekly to track their weight, set and review eating and physical activity goals, and receive personalised advice. After entering their weight and whether they had achieved the goals they had set themselves the previous week, users received tailored feedback giving encouragement if maintaining weight loss and meeting goals. Weight gain and failing to meet goals triggered automated personalised advice such as appropriate goal setting and planning, boosting motivation, overcoming difficulties and recovering from lapses.

#### Training of practice nurses

Nurses who administered child immunisations at intervention practices were trained to deliver the intervention following a standard protocol. Training took about 20–25 min to complete. Nurses were also trained in the trial procedures. Further details regarding the training of nurses have been published previously [22].

#### Usual care comparator group

Participants allocated to the usual care group received brief written information about following a healthy lifestyle at the baseline home visit.

#### Primary outcome and other outcomes

The primary aim of the trial was to assess the feasibility of undertaking a full-scale phase III cluster RCT. This was assessed via specific research questions: whether the trial was appealing to postnatal women (via assessment of the recruitment rate, to ensure a full phase III trial is feasible); whether the intervention was acceptable (via assessment of adherence to weekly self-weighing and registration with POWeR); whether the intervention had an adverse impact on child immunisation rates (recorded attendance by general practices); and the number of participants who completed the trial and the trial questionnaires (follow-up).

#### Recruitment to target

The recruitment rate is presented as a percentage based on the number of participants who took part in the trial divided by the target recruitment (*n* = 80). BWH provided data on the number of invitation letters sent.

#### Adherence and acceptability

Quantitative assessment of whether the intervention was acceptable to participants was based on the adherence to weekly self-weighing. Three sources of data regarding the frequency of self-weighing; objective recording using the BodyTrace scales, self-reported weights in the child health red book and weight recordings on POWeR were included. The objective recording of weight on the BodyTrace scales was used as the authoritative source of data to assess the frequency of self-weighing/adherence. As a secondary assessment of frequency of self-weighing and adherence, weight data from all three sources were included.

#### Use of the POWeR online weight management programme

Using participants’ email addresses, the POWeR software programme automatically recorded participants’ usage of the website (i.e. registration, number of logins, time spent on POWeR, progress through the stages, number and value of weight measurements entered).

#### Immunisation rates

To check that the intervention had no adverse impact on infant immunisation rates, practices provided data on all immunisation appointments attended during the trial. The trial took place over the first three immunisation appointments. The proportion of babies who attended all three immunisation appointments is reported.

#### Intervention fidelity

Using a tick box system, nurses were required to indicate on the weight record card whether they weighed participants at each immunisation appointment, if they asked participants if they were self-weighing each week and whether they reminded participants about using POWeR. In addition, a selection of immunisation appointments was audio recorded to assess delivery of the intervention to protocol by practice nurses. Only the parts of the consultation relevant to the intervention were recorded. These consultations were transcribed by a researcher (NTM) and then read to assess whether the nurses were delivering the intervention according to the protocol using a checklist. The checklist included that nurses weighed and recorded participants’ weight on the weight record card, checked that participants had been weighing themselves on a weekly basis, asked participants if they had accessed the POWeR website and verbally signposted participants to the POWeR website. The audio recordings were also included to allow assessment from a practical and logistical perspective on how well the intervention fitted within immunisation visits and allowed the research team to calculate how long the intervention took nurses to deliver.

#### Intervention contamination in the usual care group

The possibility of intervention contamination was assessed by asking usual care participants if they had accessed POWeR and if they knew any other women in the trial.

#### Other outcomes

Whilst this feasibility trial was not powered to detect differences in outcome measures, it provided the opportunity to ensure that there were no issues with the completion of these measures in preparation for a possible phase III trial. All measures were assessed at baseline and follow-up in both groups unless stated otherwise. Weight and percentage body fat were assessed using a Tanita SC-240MA analyser. Depression, anxiety, body image and self-reported physical activity were assessed [30–32]. Conscious cognitive energy restraint of eating, uncontrolled eating and emotional eating were assessed [33]. Weight control strategies were assessed at follow-up only [34]. Perceptions of self-weighing were assessed in the intervention group at follow-up only [35]. To inform the design of the economic evaluation in the phase III trial, we explored the acceptability (rates of completion) of the ICECAP-A instrument [36], a broader measure of wellbeing than the EQ-5D that focuses on health [37].

#### Adverse events and serious adverse events

No risks were expected to arise from taking part in the trial. The intervention was considered low risk since it only consisted of self-monitoring of weight, goal setting and using an online weight loss programme, all of which have been used in other populations and settings without evidence of harm. Therefore, adverse events were not collected. Although no serious adverse events (SAEs) were anticipated as a consequence of participation, investigators were required to report SAEs that they considered were attributable to the trial intervention.

#### Demographic, lifestyle and pregnancy-related information (both groups)

Data regarding age, ethnicity, pre-pregnancy weight, planned duration of breastfeeding, infant feeding practices, smoking, alcohol consumption, sleeping patterns, mode of delivery, pregnancy complications and how many children they had given birth to were collected at baseline. Data on timing of cessation of breastfeeding, whether participants had attended any formal weight loss programmes during their involvement in the trial and data on any specific weight loss strategies or diets that participants might have used were collected at follow-up.

#### Decision to progress to the phase III trial

For a phase III trial to take place, there needed to be evidence from this feasibility trial of meeting the pre-specified criteria (recruitment rate; adherence to weekly self-weighing and registration with POWeR) using a traffic light system [38].

- Green light: Recruitment rate of ≥ 80% of the target (*n* = 80; i.e. recruit at least 64 women), ≥50% of the intervention group weigh themselves weekly ≥60% of the time and ≥ 60% of participants have registered with the online POWeR programme. If all three criteria were met, we planned to proceed to a full trial with the protocol unchanged.
- Amber light: Recruitment rate of 50–79% of the target (*n* = 80; i.e. recruit between 40 and 63 women), 40–49% of the intervention group weigh themselves weekly 40–59% of the time and 40–59% of the intervention group registered with the online POWeR programme. If one or more of our amber-light criteria were met, we planned to adapt the protocol in light of the feedback from the interviews and our experience to improve whichever criteria has not met the ‘green-light’ level before proceeding to the full trial.
- Red light: Recruitment rate of < 50% of the target (*n* = 80; i.e. recruit less than 40 women), < 40% of the intervention group weigh themselves weekly 40–59% of the time and < 40% of the intervention group have registered with the online POWeR programme. If one or more of these criteria were met, we planned to consider the current protocol not feasible and not progress to a full RCT with the current design. It was considered important to check that the intervention did not adversely affect child immunisation rates; therefore, an additional red-light criteria was concerns that immunisation rates had been adversely affected (by comparing the rates obtained in the trial against the UK national rate).

### Trial procedures

Baseline home visits took place between 6 and 7 weeks postnatally and before the first child immunisation visit at 2 months. Participants were visited at home by a researcher where height, weight, percentage body fat were measured, BMI calculated; eligibility (inclusion/exclusion criteria) was reviewed; informed consent was obtained for eligible participants and the baseline health questionnaire booklet was completed/collected. Participants were informed which group they were allocated to in the trial. The usual care group were issued with the healthy lifestyle leaflet and advised that they would receive usual care at their child immunisation appointments. The intervention group were issued with the healthy lifestyles leaflet; the weight record card was attached to red immunisation book; a trial sticker was placed on the front of the red book and participants were given BodyTrace scales, instructed on use (issued instruction leaflet) and provided with instructions and individual login details for POWeR.

Follow-up visits took place 3 months after participants entered the trial. Participants were visited at home by a member of the research team and the following tasks were completed: weight and percentage body fat measured, BMI calculated, follow-up questionnaires collected. Questionnaires were posted to participants 5–7 days in advance (for collection by the researcher); confirmation of attendance at immunisation appointments was obtained. Participants’ weight record cards were collected. A £20 shopping voucher was offered to all participants at follow-up as reimbursement for any inconvenience trial participation may have caused them.

### Sample size

As this was a feasibility trial, a formal sample size calculation was not conducted. The trial was not designed or powered to detect a statistically significant difference in efficacy between the two trial groups. A sample size of at least 70 participants has been recommended for pilot trials; therefore, recruitment of a sample of 80 participants from 10 to 12 practices recruited over 8 months was set [39].

### Data analysis

#### Analysis of outcome measures

The recruitment rate is calculated as a percentage based on the number of participants who took part in the trial divided by the target recruitment (*n* = 80). The percentage of participants in the intervention group who adhered to weekly self-weighing and who registered with POWeR is also presented. The binomial normal approximation was used to calculate the corresponding 95% confidence intervals (CI). All primary analyses of outcome data were by intention-to-treat. The primary comparison groups involved those in the weight management intervention group versus the usual care group. The analysis of outcome data focused on confidence interval estimation. Continuous outcomes (except the Pregnancy Physical Activity Questionnaire; see below) were summarised using means and standard deviations. Adjusted mean differences between groups and the corresponding 95% CIs were estimated from generalised linear mixed models which included adjustment for baseline values (where available) and the minimisation variables (general practice size and index of multiple deprivation), and practice (cluster) as a random effect. The Pregnancy Physical Activity Questionnaire (PPAQ) was highly skewed (using the Shapiro-Wilk test for normality) for a number of domains, and so the PPAQ scores were summarised using medians with interquartile ranges (IQR). The unadjusted difference between the median in each group was reported along with 95% CIs calculated using bootstrapping methods [40]. All estimates of differences between groups are presented with two-sided 95% CI and no *p* values are presented.

Use of POWeR was assessed through the number of times participants logged on to POWeR, recorded their weight on POWeR and time spent browsing, with data presented as medians with interquartile ranges. These data were also tabulated at each intervention week to assess usage over time. Progress through POWeR was assessed by tabulating the number of stages participants completed and the number of participants who completed each stage.

## Results

### Recruitment of practices and participants

Fourteen practices were recruited to participate; seven were randomised to deliver the weight management intervention and seven to deliver usual care. A total of 368 invitation letters were sent by BWH to potentially eligible women from participating practices. Twenty-eight women consented to participate between July 2018–April 2019 (10 months) at an average rate of 2.8 participants per month. Sixteen participants were registered at practices who delivered the weight management intervention and 12 participants at practices that delivered usual care. For intervention and usual care practices, the number of participants recruited ranged between one and five and zero and eight respectively. Trial follow-up was completed in September 2019.

### Participant trial flow

**Fig. 1:**
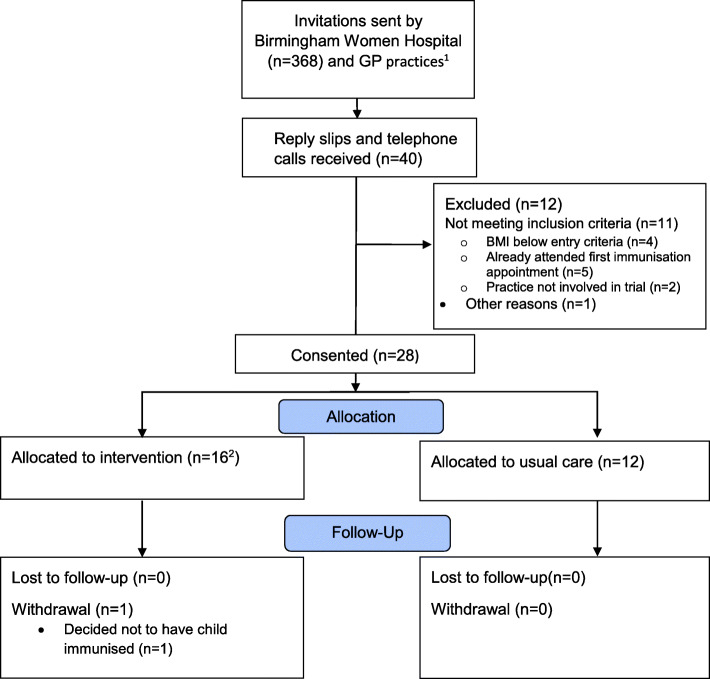
Participant flow through the trial

Figure 1 shows participant flow through the trial. The most common reason for non-recruitment was related to potentially eligible women having already attended their first child immunisation appointment (*n* = 5) or having a BMI below 25 kg/m^2^ (*n* = 4).

### Participant characteristics at baseline

The average age of participants was 32.1 years (SD = 5.7). Forty-six percent of participants (*n* = 13) were of White ethnicity. Most participants were married or living with their partner (74%, *n* = 20). The average weight and BMI of participants at baseline was 83.6 kg (SD = 17.1) and 31.8 kg/m^2^ (SD = 6.9) respectively. Most participants had given birth to two children (43%, *n* = 12). Fifteen participants (53%) were exclusively breast feeding. See Table 1.

**Table 1:** Baseline characteristics by trial group

Demographic and other baseline variables	Intervention (***N*** = 16)	Usual care (***N*** = 12)	Overall (***N*** = 28)
Age (years)			
Mean (SD, *N*)	32.9 (6.1, 16)	31.0 (5.3, 12)	32.1 (5.7, 28)
Min-max	22–41	24–42	22–42
Ethnic group			
White	5 (31%)	8 (67%)	13 (46%)
Pakistani	3 (19%)	0 (0%)	3 (11%)
Other Asian	3 (19%)	0 (0%)	3 (11%)
Black Caribbean	0 (0%)	1 (8%)	1 (3%)
Black African	1 (6%)	2 (17%)	3 (11%)
Other	4 (25%)	1 (8%)	5 (18%)
Current marital status			
Single (living alone)	1 (6%)	3 (27%)	4 (15%)
Single (living with partner)	3 (19%)	3 (27%)	6 (22%)
Married	11 (69%)	3 (27%)	14 (52%)
Divorced/separated (living alone)	0 (0%)	1 (9%)	1 (4%)
Other^1^	1 (6%)	1 (9%)	2 (7%)
Missing	0	1	1
Current employment status			
In paid employment	9 (56%)	6 (55%)	15 (56%)
Unemployed	1 (6%)	2 (18%)	3 (11%)
Student	1 (6%)	0 (0%)	1 (4%)
Looking after the home/family	5 (31%)	1 (9%)	6 (22%)
Sick/disabled	0 (0%)	1 (9%)	1 (4%)
Other^2^	0 (0%)	1 (9%)	1 (4%)
Missing	0	1	1
Current financial status			
Normally have enough money	9 (56%)	0 (0%)	9 (35%)
Enough money if I plan carefully	6 (38%)	4 (40%)	10 (38%)
Enough money for basic things	1 (6%)	3 (30%)	4 (15%)
Basic things hard to afford	0 (0%)	3 (30%)	3 (12%)
Missing	0	2	2
Average number of cigarettes smoked each day			
None	16 (100%)	9 (90%)	25 (96%)
5 or less	0 (0%)	0 (0%)	0 (0%)
6–10	0 (0%)	1 (10%)	1 (4%)
Missing	0	2	2
Drank alcohol in last week			
Yes	4 (25%)	2 (18%)	6 (22%)
No	12 (75%)	9 (82%)	21 (78%)
Mean number of units			
Mean (SD, *N*)	5.7 (3.5, 3)	4.0 (2.8, 2)	5.0 (3.0, 5)
Min-max	2–9	2–6	2–9
Missing	1	0	1
Weight (kg)			
Mean (SD, *N*)	81.6 (13.7, 16)	86.2 (21.2, 12)	83.6 (17.1, 28)
Min-max	58.8–106.7	66.7–148.8	58.8–148.8
BMI (kg/m^2^)			
Mean (SD, *N*)	31.6 (6.1, 16)	32.1 (8.0, 12)	31.8 (6.9, 28)
Min-max	25.5–47.2	26.3–56.4	25.5–56.4
Percentage body fat (%)			
Mean (SD, *N*)	40.9 (4.0, 16)	41.6 (6.0, 12)	41.2 (4.8, 28)
Min-max	34.6–46.8	32.5–56.1	32.5–56.1
**Pregnancy details**			
Weight before pregnancy (kg)			
Mean (SD, *N*)	75.0 (14.6, 13)	83.9 (31.0, 10)	78.9 (23.0, 23)
Missing	3	2	5
Number of children given birth to			
1	6 (37%)	1 (8%)	7 (25%)
2	7 (44%)	5 (42%)	12 (43%)
≥3	3 (19%)	6 (50%)	9 (32%)
Number of children living in household			
1	6 (37%)	1 (8%)	7 (25%)
2	7 (44%)	5 (42%)	12 (43%)
≥3	3 (19%)	6 (50%)	9 (32%)
Complications during this pregnancy			
Yes	8 (50%)	3 (25%)	11 (39%)
No	8 (50%)	9 (75%)	17 (61%)
*If yes (n = 11, not mutually exclusive):*			
Gestational diabetes	1 (12.5%)	2 (67%)	3 (27%)
Pre-eclampsia	1 (12.5%)	0 (0%)	1 (9%)
Gestational hypertension	4 (50%)	0 (0%)	4 (36%)
Pre-term delivery	1 (12.5%)	0 (0%)	1 (9%)
Neonatal intensive care/special care	1 (12.5%)	0 (0%)	1 (9%)
Other^3^	2 (25%)	2 (67%)	4 (36%)
Type of delivery			
Normal vaginal delivery	10 (63%)	8 (67%)	18 (64%)
Instrumental vaginal delivery	1 (6%)	0 (0%)	1 (4%)
Elective caesarean section	1 (6%)	1 (8%)	2 (7%)
Emergency caesarean section	4 (25%)	3 (25%)	7 (25%)
**Pregnancy and breastfeeding**			
Tried to breastfeed baby			
Yes	16 (100%)	11 (92%)	27 (96%)
No	0 (0%)	1 (8%)	1 (4%)
Current method of feeding			
Exclusively breastfeeding	11 (69%)	4 (33%)	15 (53%)
Exclusively formula feeding	3 (19%)	5 (42%)	8 (29%)
Both breastmilk and formula	2 (12%)	3 (25%)	5 (18%)
*If breastfeeding:*	*(n = 13)*	*(n = 7)*	*(n = 20)*
Intended time to continue breastfeeding			
Up to 3 months	0 (0%)	0 (0%)	0 (0%)
Up to 6 months	2 (15%)	1 (14%)	3 (15%)
Up to 9 months	1 (8%)	0 (0%)	1 (5%)
Up to 12 months	1 (8%)	1 (14%)	2 (10%)
> 1 year	5 (38%)	3 (43%)	8 (40%)
As long as possible	4 (31%)	2 (29%)	6 (30%)
**Sleep**			
Average amount of uninterrupted sleep per night (hours)			
Mean (SD, *N*)	3.0 (1.2, 16)	3.5 (0.9, 12)	3.2 (1.1, 28)
Min-max	1–6	2–5	1–6

^1^Others (*n* = 2): living with partner; not living with partner. ^2^Others (*n* = 1): In paid employment and a student. ^3^Others (*n* = 4): pelvic pain (on crutches from 37/40), hyperemesis and excess water, hyperemesis and high-risk embolism/thrombosis

### Adherence to self-weighing (intervention group)

Most participants (62.5%, *n* = 10) weighed themselves in at least eight of the weeks over the follow-up period. A total of 62.5% (*n* = 10) weighed themselves using the Body Trace scales at least 60% of the time, zero percentage between 40 and 59% of the time and 37.5% (*n* = 6) less than 40% of the time. When weight data from all three sources were used, 69% (*n* = 11) weighed themselves at least 60% of the time, 6% (*n* = 1) between 40 and 59% and 25% (*n* = 4) less than 40% of the time.

### Use of the POWeR online weight management programme (intervention group)

A total of 9/16 (56%) of the intervention group registered to use the POWeR online programme (objective data). The median number of times these participants logged onto POWeR over the intervention period was 4 (IQR 2–9, range 1–21). Registered participants recorded their weight on POWeR a median of two times (IQR 1–7) and spent a median of 102.8 min in total on POWeR over the intervention period (IQR 58.4–189.4). Four of the nine (44%) participants who registered on POWeR completed stage 1 and two (22%) completed stage 2. No participants completed all three stages.

### Stop-go criteria to proceed to a phase III trial

Twenty-eight participants (from a planned recruitment of 80 participants; 35% of target) consented to the trial; therefore, the recruitment target was not met (red) (95% CI 25 to 45%). Registration with the POWeR website was categorised as amber as 56% (9/16) of participants registered with the programme (95% CI 32 to 81%). The stop-go criteria for adherence to weekly self-weighing were met (green) with 63% (10/16) of participants achieving this target (95% CI 39 to 86%).

### Acceptability of the intervention to participants

The intervention group were asked a series of questions to assess their views on the acceptability of the study/intervention where response scores could range from 1 to 8 (higher scores were more favourable). The mean score for the question ‘would you recommend this study to your friends?’ was 6.2/8. On average, the usefulness of being weighed by the practice nurse was scored 5.3/8 and usefulness of weekly self-weighing scored 5.8/8 by participants. Overall, participants felt it was appropriate for the nurse to weigh them at child immunisation visits (average score 6.1/8). To assess the impact of the intervention on participants’ psychological health, a question that assessed whether the intervention made participants feel anxious about their weight was included; average score was 3.8/8.

### Intervention fidelity assessed by audio recordings of consultations

A total of 17 (from a possible 45) audio recordings from immunisation appointments were recorded, involving 10 participants from six intervention practices. The aim was to audio record as many appointments as possible and this data reflects those where both participants and nurses consented to having the consultation audio recorded. Data from the audio recordings indicated that the intervention took less than 2 min to deliver in eleven consultations, between 2 and 3 min in five consultations and between 3 and 4 min in one consultation. The results show evidence of a high level of intervention fidelity by practice nurses against the intervention checklist (Table 2).

**Table 2:** Results from audio recordings of intervention consultations

	Completed by nurse	Not completed by nurse	Not clear from recording^**1**^	N/A
Weighed and recorded weight in child health red book	15 (88.2%)	1 (5.9%)	0	1 (5.9%)
Checked participant was weighing weekly	15 (88.2%)	2 (11.8%)	0	0
Asked if accessed the POWeR website	15 (88.2%)	1 (5.9%)	1 (5.9%)	0
Signposted to POWeR	13 (76.5%)	3 (17.6%)	1 (5.9%)	0

^1^Not clear—did not hear direct evidence of this in the audio recording but this task may have been completed after the recorder was switched off

### Delivery of the intervention by practice nurses

Weight record cards were available for 12/16 intervention group participants. Delivery of the intervention components by practice nurses was high across all immunisation appointments. See Table 3 for data.

**Table 3:** Delivery of the intervention by nurses at immunisations

	Intervention (***N*** = 12)^**1,2**^
**2-month immunisation appointment**	
Appointment attended by participant	
Yes	12 (100%)
No	0 (0%)
When participant weighed in relation to the immunisation of the child	
Before	5 (71%)
After	2 (29%)
Declined	0 (0%)
Missing	5
Weight recorded by nurse at immunisation appointment	
Yes	12 (100%)
No	0 (0%)
Participant reminded by nurse about POWeR	
Yes	12 (100%)
No	0 (0%)
Participant asked by nurse if following weekly self-weighing	
Yes	12 (100%)
No	0 (0%)
Missing	0 (0%)
**3-month immunisation appointment**	
Appointment attended by participant	
Yes	11 (100%)
No	0 (0%)
Missing	1^3^
When participant weighed in relation to the immunisation of the child	
Before	4 (100%)
After	0 (0%)
Declined	0 (0%)
Missing	8
Weight recorded by nurse at immunisation appointment	
Yes	11 (100%)
No	0 (0%)
Missing	1
Participant reminded by nurse about POWeR	
Yes	11 (100%)
No	0 (0%)
Missing	1
Participant asked by nurse if following weekly self-weighing	
Yes	11 (100%)
No	0 (0%)
Missing	1
**4-month immunisation appointment**	
Appointment attended by participant	
Yes	10 (100%)
No	0 (0%)
Missing	2^4^
When participant weighed in relation to the immunisation of the child	
Before	2 (100%)
After	0 (0%)
Declined	0 (0%)
Missing	10
Weight recorded by nurse at immunisation appointment	
Yes	10 (100%)
No	0 (0%)
Missing	2
Participant reminded by nurse about POWeR	
Yes	9 (100%)
No	0 (0%)
Missing	3
Participant asked by nurse if following weekly self-weighing	
Yes	9 (100%)
No	0 (0%)
Missing	3

^1^One participant in the intervention group withdrew prior to follow-up visit which is when the weight record card is collected. ^2^Three of the 15 participants who reached the end of the trial returned a completely blank weight record card. ^3^Appointment was attended by participant according to follow-up form and GP records. ^4^Appointment was attended by participant (*n* = 1) and grandparent (*n* = 1) according to follow-up form and GP records

### Attendance at immunisation visits (data from medical records)

Practices provided immunisation data on 24 participants (expected data on 27 participants, as one woman in the intervention group withdrew from the trial as they decided not to have their child immunised). There was no evidence that the intervention deterred participants from attending their child immunisation appointment, with 12/13 participants (92%) in the intervention group for whom this data was provided attending all three child immunisation appointments and having their baby immunised.

### Intervention contamination (usual care group)

Only one usual care participant reported using portion control methods to help them lose weight and none reported accessing POWeR or any other online weight loss programme. Three usual care participants reported knowing someone else taking part in the study.

### Clinical and participant reported outcomes

#### Body composition, psychological health and other outcomes

The usual care group was 7.5 kg (based on adjusted mean difference) heavier in weight than the intervention group at follow-up (95% CI − 13.8 to − 1.3). The within group profile of weight over time showed that the intervention group on average lost weight (unadjusted mean change: − 3.3 kg), whilst the usual care group gained weight (unadjusted mean change: + 1.9 kg). The intervention group had lower BMI and percentage body fat scores than usual care at follow-up (see Table 4). The intervention group reported higher anxiety scores (adjusted mean difference = 3.7, 95% CI 0.9 to 6.4) and marginally higher depression scores (adjusted mean difference = 0.5; 95% CI − 1.9 to 2.9) at follow-up than the usual care group (see Table 5). The intervention group reported a more favourable body image score than usual care at follow-up (adjusted mean difference = 0.9, 95% CI − 0.5 to 2.4) (see additional file 1). No serious adverse events were reported. Rates of breastfeeding were higher in the intervention group (67%, *n* = 10) than for usual care (33%, *n* = 4) at follow-up.

**Table 4:** Body composition

	Baseline		3-month follow-up		
	Intervention (***N*** = 16)	Usual care (***N*** = 12)	Intervention (***N*** = 15)^**1**^	Usual care (***N*** = 12)	Adjusted mean difference^**2**^ (95% CI)
Weight (kg)					
Mean (SD, *N*)	81.6 (13.7, 16)	86.2 (21.2, 12)	78.3 (13.5, 15)	88.1 (23.9, 12)	−7.5 (−13.8, −1.3)
Min-max	58.8–106.7	66.7–148.8	60.5–106.7	64.1–154.3	
% Body fat					
Mean (SD, *N*)	40.9 (4.0, 16)	41.6 (6.0, 12)	39.6 (4.7, 15)	42.4 (7.1, 12)	−3.2 (−6.3, −0.1)
Min-max	34.6–46.8	32.5–56.1	34.5–48.9	30.5–57.0	
BMI^3^					
Mean (SD, *N*)	31.6 (6.1, 16)	32.1 (8.0, 12)	30.2 (6.0, 15)	32.8 (8.8, 12)	−3.1 (−5.8, −0.3)
Min-max	25.5–47.2	26.3–56.4	24.5–47.2	24.0–58.4	
BMI category^3^					
Healthy (18.5–24.9)	0 (0%)	0 (0%)	2 (13%)	1 (8%)	
Overweight (25–29.9)	8 (50%)	6 (50%)	8 (53%)	5 (42%)	
Obese (30–39.9)	6 (37.5%)	5 (42%)	4 (27%)	5 (42%)	
Morbidly obese (> 40)	2 (12.5%)	1 (8%)	1 (7%)	1 (8%)	

^1^One intervention group participant withdrew prior to follow-up. ^2^Values < 0 favour the intervention. Adjusted for practice (random effect), the two minimisation variables (GP size list and index of multiple deprivation), and baseline value for each outcome. ^3^BMI at 3-month follow-up calculated using the height recorded at baseline and 3-month follow-up weight

**Table 5:** Anxiety and depression

	Baseline		3-month follow-up		
	Intervention (***N*** = 16)	Usual care (***N*** = 12)	Intervention (***N*** = 15^**1**^)	Usual care (***N*** = 12)	Adjusted mean difference^**2**^ (95% CI)
HADS: Depression^3^					
Mean (SD, *N*)	5.9 (4.9, 16)	5.0 (3.0, 11)	6.3 (4.0, 14)	5.5 (2.5, 12)	0.5 (− 1.9, 2.9)
Minimum-maximum	0–15	1–10	0–15	2–10	
Missing	0	1	1	0	
HADS Anxiety					
Mean (SD, *N*)	6.1 (3.8, 16)	6.3 (3.7, 11)	8.4 (4.1, 14)	5.2 (3.6, 12)	3.7 (0.9, 6.4)
Minimum-maximum	0–11	1–13	1–14	1–13	
Missing	0	1	1	0	

^1^One intervention group participant withdrew prior to follow-up. ^2^Values < 0 favour intervention. Adjusted for GP practice (random effect), the two minimisation variables (GP size list and index of multiple deprivation), and baseline score. ^3^HADS domain scores range from 0 to 21, where higher scores indicate more severe anxiety/depression

#### Self-reported physical activity and sedentary behaviour

Data from the PPAQ was skewed and so is reported using medians and unadjusted differences in medians are reported. The intervention group reported participating in more moderate-intensity physical activity at follow-up (difference = 22.3 MET hours/week: 95% CI − 71.4 to 116.0), but less light-intensity physical activity (difference = − 19.6 MET hours/week: 95% CI − 84.9 to 45.7) than usual care. The intervention group spent more time sedentary than usual care (difference = 8.4 MET hours/week: 95% CI − 23.7 to 40.5) (see Table 6).

**Table 6:** Physical activity and sedentary behaviour

	Baseline		3-month follow-up		
	Intervention (***N*** = 16)	Usual care (***N*** = 12)	Intervention (***N*** = 15)^**1**^	Usual care (***N*** = 12)	Difference in Medians^**2**^ (95% CI)
**Intensity domains**					
PPAQ: Sedentary activity (MET hours/week)					
Median [IQR]	60.2 [30.6, 88.5]	66.0 [28.0, 97.1]	48.1 [21.0, 56.9]	39.7 [22.4, 60.8]	8.4 (−23.7, 40.5)
Minimum-maximum	9.5–108.2	22.4–146.3	5.1–98.7	11.6–109.9	
Missing	2	1	2	0	
PPAQ: Light-intensity activity (MET hours/week)					
Median [IQR]	119.2 [87.7, 154.9]	154.9 [135.5, 166.1]	110.8 [81.2, 178.0]	130.4 [107.8, 183.9]	−19.6 (−84.9, 45.7)
Minimum-maximum	49.5–198.1	110.4–206.3	48.8–193.6	73.9–229.8	
Missing	1	1	2	1	
PPAQ: Moderate-intensity activity (MET hours/week)					
Median [IQR]	116.4 [58.5, 168.6]	141.5 [70.8, 188.7]	150.8 [82.3, 199.4]	128.5 [56.5–167.0]	22.3 (−71.4, 116.0)
Minimum-maximum	10.6–210.4	58.6–206.5	50.2–266.1	55.1–361.3	
Missing	0	1	1	1	
PPAQ: Vigorous-intensity activity (MET hours/week)					
Median [IQR]	0 [0, 5.8]	0 [0, 0]	1.6 [0, 9.8]	3.3 [0, 7.5]	−1.6 (−8.2, 4.9)
Minimum-maximum	0–9.8	0–30.0	0–10.1	0–37.0	
Missing	0	1	1	0	
**Activity type domains**					
PPAQ: Household/caregiving activity (MET hours/week					
Median [IQR]	202.6 [121.8, 241.5]	224.2 [169.6, 272.8]	181.0 [134.4, 283.6]	219.5 [150.9, 309.1]	−38.5 (− 159.3, 82.3)
Minimum-maximum	22.4–371.4	146.5–353.9	72.5–425.8	119.6–401.8	
Missing	1	1	2	0	
PPAQ: Occupational activity (MET hours/week)					
Median [IQR]	0 [0, 69.9]	0 [0, 71.1]	0 [0, 35.9]	0 [0, 18.0]	0 (−19.9, 19.9)
Minimum-maximum	0–138.8	0–158.6	0–239.8	0–109.4	
Missing	0	1	0	1	
PPAQ: Sports/exercise activity (MET hours/week)^3^					
Median [IQR]	6.3 [0, 20.3]	2.4 [0.8, 22.0]	18.0 [5.3, 29.6]	9.1 [6.4, 17.0]	8.9 (−5.0, 22.8)
Minimum-maximum	0–34.2	0–53.4	0–43.2	2.3–37.8	
Missing	0	1	1	0	
**Total activity**					
PPAQ: Total activity (MET hours/week)					
Median [IQR]	289.7 (224.2, 416.2)	345.6 [328.1, 421.3]	265.4 [224.8, 434.6]	278.6 [212.8, 409.7]	−13.2 (−209.1, 182.7)
Minimum-maximum	114.2–456.9	265.4–438.6	123.2–498.6	178.0–652.8	
Missing	3	1	3	1	
PPAQ: Total activity (excluding work domain^4^) (MET hours/week)					
Median [IQR]	289.7 (181.0, 323.0)	328.1 [271.1, 362.4]	237.3 [178.9, 396.2]	301.5 [217.0, 401.4]	−64.2 (− 213.1, 84.6)
Minimum-maximum	71.2–456.9	261.0–423.6	123.2–498.6	178.0–543.4	
Missing	3	1	3	0	

^1^One participant in the intervention group withdrew prior to follow-up. ^2^Values > 0 favour intervention, except for PPAQ: Sedentary activity where values < 0 favour intervention.^3^ Three participants at baseline and two at follow-up indicated that they had done something else for exercise but did not indicate what this exercise was. It is assumed that the unspecified exercises undertaken had a moderate intensity (MET value = 4.45). Sensitivity analysis performed assuming that these exercises were of low intensity (MET value = 2.9) and vigorous intensity (MET value = 6) gave very similar results to those presented here. ^4^Work domain (questions 33–37 only answered by those in work at the time of completion) are excluded here

#### Eating behaviours

The intervention group reported better cognitive restraint of eating and uncontrolled eating scores than the usual care group at follow-up. The usual care group reported better emotional eating scores than the intervention group at follow-up (see Table 7).

**Table 7:** Eating behaviour

	Baseline		3-month follow-up		
	Intervention (***N*** = 16)	Usual care (***N*** = 12)	Intervention^**1**^ (***N*** = 15)^**1**^	Usual care (***N*** = 12)	Adjusted mean difference^**2**^ (95% CI)
TFEQ: Cognitive restraint domain^3^					
Mean (SD, *N*)	38.7 (15.0, 16)	44.1 (28.1, 11)	47.6 (12.7, 14)	48.6 (23.3, 12)	5.4 (−8.9, 19.6)
Minimum-maximum	16.7–66.7	11.1–77.8	22.2–72.2	0–72.2	
Missing	0	1	1	0	
TFEQ: Uncontrolled eating domain					
Mean (SD, *N*)	47.9 (26.3, 16)	43.0 (23.7, 11)	50.3 (25.6, 14)	41.0 (27.9, 12)	−0.03 (−15.4, 15.4)
Minimum-maximum	7.4–88.9	7.4–88.9	14.8–85.2	3.7–81.5	
Missing	0	1	1	0	
TFEQ: Emotional eating domain					
Mean (SD, *N*)	47.9 (26.5, 16)	48.5 (32.7, 11)	56.3 (34.4, 14)	43.5 (32.3, 12)	9.1 (−25.9, 44.0)
Minimum-maximum	0–100	0–88.9	0–100	0–88.9	
Missing	0	1	1	0	

^1^One intervention group participant withdrew prior to follow-up. ^2^Values < 0 favour intervention, except for the cognitive restraint domain where values > 0 favour intervention. Adjusted for GP practice (random effect), the two minimisation variables (GP size list and index of multiple deprivation), and baseline score. ^3^TFEQ domain scores range from 0 to 100, where higher scores indicate more positive behaviour in the cognitive restraint domain and higher scores indicate more negative behaviour in the uncontrolled eating and emotional eating domains

#### Weight control strategies and perceptions of self-weighing (intervention group)

At follow-up, average scores for engagement in individual item weight control strategies were comparable across the groups (see additional file 2). Overall, participants in the intervention group reported positive perceptions of regular self-weighing at 3 months with an average score of 5.1/8. Individual item scores for perceptions of self-weighing ranged from 4.2 to 5.8/8 (see additional file 3).

#### Withdrawals, loss to follow-up and missing data

One participant withdrew from the trial as she decided not to have her child immunised. There were no losses to follow-up. Further information regarding withdrawals, loss to follow for each outcome and missing data is reported in additional file 4.

## Discussion

This study examined the feasibility and acceptability of a multi-component brief weight management intervention delivered to women at child immunisation appointments. The recruitment target was not met (red) and changes to the methods of recruitment are required before proceeding to a phase III trial. The target for adherence to regular self-weighing was met (green), albeit with wide confidence intervals in part due to the small sample size. Participants regularly recorded their weight on the weight record card, demonstrating that they adhered well to the main intervention component. The stop-go criteria for use of the POWeR website was categorised as amber; therefore, some additional strategies may be needed to encourage participants to engage with an online weight management programme and to maintain adherence over time. No participants were lost to follow-up. There was also a signal from the follow-up weight data that the intervention may help participants to lose weight. Furthermore, the increase in weight in the usual care group highlights the importance of encouraging action to prevent additional weight gain during the postnatal period. The intervention did not have an adverse effect on attendance at immunisation appointments. The intervention took practice nurses on average 2 min to deliver, and intervention fidelity by nurses was high suggesting that the intervention can be delivered within child immunisations in primary care. Missing data for questionnaire-based outcomes was low, ranging from 0% to 15.

### Recruitment

Slower than expected recruitment rates are not uncommon in postnatal weight management studies [41, 42]; recruitment also proved challenging in this trial for which there may be specific reasons. All practices except one were located in areas of high deprivation serving a high proportion of ethnic minority patients. The number of participants who reported ‘difficult financial status’ was high (65%) and a high proportion were from non-White ethnicities (54%). Recruitment to clinical trials from these populations is known to be difficult; therefore, our recruitment experiences are likely to represent a ‘worst-case scenario’ [43]. Recruitment was also hampered by a change in the computer system at BWH in the final 6 weeks of recruitment; this made it difficult for the hospital to systematically identify potentially eligible women.

Participants received the study invitation letter around 4–6 weeks after giving birth. This is a time in which new mothers are adjusting to life with a small baby; therefore, weight loss may not be considered a priority at this time. There was a maximum period of 4 weeks available between women receiving their invitation letter and being able to complete the baseline assessment of outcomes. Women could not be recruited prior to 4 weeks postnatally, and the baseline visit had to be completed before the first immunisation. This short time period may have deterred some women from participating at this busy time in their lives. An alternative approach may be to consider recruiting women antenatally towards the end of pregnancy, when women do not have the same distractions and demands on their time. Given the short window of opportunity available to recruit women, it may be that an ‘opt-out’ approach to recruitment would be more fruitful. Whilst accepting the ethical challenges that might occur from such an approach, evidence has suggested that higher response and recruitment rates may be obtained when studies employ opt-out methods [44, 45] and data from trials offering weight management in routine care show that the overwhelming majority of people who are obese consider this to be helpful and appropriate, whether or not they take up the offer of support [46]. Considering the recruitment response to this trial, it is also possible that women do not want a weight management intervention shortly after giving birth, although this is not consistent with evidence that has reported women do want early intervention [47, 48].

### Adherence to self-weighing

Regular self-weighing has been shown to be an important strategy in facilitating weight loss, particularly within multi-component weight loss interventions [35, 49]. Here, adherence to weekly self-weighing was good, demonstrating that women are keen to engage in weight related self-management behaviours. Data collected via the objective recording Body Trace scales showed that 63% of the intervention group weighed themselves weekly ≥ 60% of the time, meeting the green stop-go criteria. These data show that at least some postnatal women are motivated to engage in regular self-weighing soon after childbirth, a strategy that has been shown to be instrumental in facilitating weight loss in other populations [50–54]. One of the attractions of self-management-based interventions is that they are flexible, individualised and can be engaged in by women at a time that suits their busy daily lives. Strategies to enhance this outcome could be considered to further increase engagement with this behaviour. Technology such as text message reminders or opportunities to track their weight change may be useful.

### Use of the POWeR online weight management programme

A total of 56% of participants registered to use the POWeR website and the amber stop-go criteria for progression was met. To have met the green stop-go criteria, ≥ 60% of participants needed to have registered with POWeR, highlighting that prior to a subsequent phase III trial, women may benefit from some additional support in using technology to support their weight loss efforts. It might also be the case that well-known branded online weight loss programmes (e.g. Weight Watchers or Slimming World) may be more appealing. The low number of times participants recorded a weight in POWeR is likely to be related to the weight record card being used instead. As weights on the record card were reviewed at the immunisation appointment (external accountability), participants were more likely to record their weight on the card, rather than using POWeR. Engagement with POWeR reduced over time, although this programme was designed to not require intensive use over time. Studies have shown that use of additional sessions after the core sessions is not related to additional weight loss [55]. Nevertheless, any future digital programme would benefit from strategies to enhance effective engagement.

### Delivery of the intervention by nurses at child immunisation appointments

In the UK, guidelines advise health care professionals to screen for obesity and encourage weight loss via the provision of information and signposting to available weight management services [27, 56]. Yet evidence has shown that health professionals are reluctant to raise the topic of weight with patients for fear of negative consequences such as causing offence and upsetting patients [57]. This trial has provided data to show that practice nurses were able to ‘raise the topic of weight’ and deliver the intervention per protocol. Nurses delivered all components of the intervention with high fidelity. Audio recordings of the immunisation appointments demonstrated that overall nurses delivered the intervention well, and according to the protocol, providing reassurance that the nurse training methods worked well and that the intervention can be delivered as intended within child immunisations.

### Strengths and limitations

This study has several methodological strengths and makes a unique contribution to the literature in several ways. This is the first study worldwide to assess the merits of a weight loss intervention embedded within a national child immunisation programme. This study was equally appealing to both first-time and multiparous women, highlighting that weight management during the postnatal period is a concern to women irrespective of the number of children they have given birth to. Whilst the recruited sample was small, women varied in terms of their socio-economic status, ethnicity and employment status, suggesting that the experiences of a wide range of women are represented in the findings. Importantly, the sample included a high proportion of women from more deprived areas and ethnic groups. Practice nurses were trained to deliver the intervention following standardised procedures ensuring that the intervention had the best opportunity to be successful; evidence shows nurses adhered well to the protocol.

Process evaluations are often not included when evaluating complex health behaviour change interventions. Several approaches to process evaluation were included in this trial in relation to its setting, intervention delivery and the acceptability and implementation of the intervention. A selection of immunisation appointments during which the intervention was delivered were audio recorded and this provided objective ‘real-time’ data on the interactions between participants and nurses to further enhance our understanding of how the intervention could be refined to maximise its effectiveness. The inclusion of BodyTrace weighing scales allowed objective data on the frequency with which participants weighed themselves to be collected, providing further real-time objective process evaluation data.

Assessments of weight were objectively measured by a researcher to ensure these data were accurate to minimise missing data. Weight loss studies can often experience high loss to follow-up rates, but we were able to collect weight data on all participants who completed follow-up (27/28; 1 participant withdrew). Objective data on attendance at immunisations were collected from medical records. This study provides reassurance that the intervention would be unlikely to adversely impact immunisation rates, which is critical to the safety of the intervention.

This study should also be interpreted in the light of some methodological limitations. By using a centralised hospital records system to invite all women who had given birth to take part, the aim was to reduce the likelihood of recruiting highly motivated women, but we cannot discount the possibility that atypical women were recruited. As this was a feasibility trial, the sample size was small, and the findings should be interpreted with this in mind. Participants self-reported their physical activity levels, and future studies should include an objective assessment. The intervention was assessed over the first three immunisation appointments at 1, 3 and 4 months, so the longer-term effects of the intervention were not assessed.

## Conclusions

This trial has provided evidence that a brief weight loss intervention that promotes self-management of weight delivered by nurses within routine child immunisations visits was acceptable to women recruited in this trial. Nurses were able to deliver the intervention with high fidelity indicating the intervention was feasible to deliver within child immunisation appointments. Adherence to weekly self-weighing was generally good. Uptake of the online weight management programme was acceptable but there is scope for improvement. However, recruitment was a challenge, and the methods used to recruit postnatal women were not successful. Alternative approaches need to be tested prior to progressing to a phase III trial.

## Supplementary information

**Additional file 1.** Body image.

**Additional file 2.** Weight control strategies.

**Additional file 3.** Perceptions of self-weighing.

**Additional file 4.** Withdrawals, loss to follow-up and missing data.

## Data Availability

The datasets used and analysed during this study are available from the corresponding author on reasonable request. Access to anonymised data may be granted following review of the request. Exclusive use will be retained until the publication of major outputs.

## Abbreviations

AE: Adverse events
BMI: Body mass index
BWH: Birmingham Women’s Hospital
CI: Confidence interval
RCT: Randomised controlled trial
SAE: Serious adverse events
POWeR: Positive Online Weight Reduction
