# Supplementary material for: Feasibility and acceptability of a brief routine weight management intervention for postnatal women embedded within the national child immunisation programme in primary care: randomised controlled cluster feasibility trial
Source: Trials. 2020 Sep 1;21:757. doi: 10.1186/s13063-020-04673-9 (PMC7466790; doi:10.1186/s13063-020-04673-9)
Supplement: Supplementary file 1 — Additional file 1. Body image. [file 13063_2020_4673_MOESM1_ESM.docx]

**Additional file 1.**

| BISS: overall measure^3^ | **Baseline** | | **3-month follow-up** | | |
| --- | --- | --- | --- | --- | --- |
|  | **Intervention**  **(N=16)** | **Usual Care**  **(N=12)** | **Intervention**  **(N=15)^1^** | **Usual Care**  **(N=12)** | **Adjusted mean dfference^2^ (95% CI)** |
| Mean (SD, N) | 3.5 (0.9, 15) | 3.3 (1.0, 11) | 3.9 (1.4, 13) | 3.0 (1.5, 12) | 0.9 (-0.5, 2.4) |
| Min-Max | 1.8-4.8 | 1.8-4.8 | 1.2-6.0 | 1.0-5.2 |  |
| Missing | 1 | 1 | 2 | 0 |  |

^1^One intervention group participant withdrew prior to follow-up. ^2^Values >0 favour intervention. Adjusted for practice (random effect), the two minimisation variables (practice list size and index of multiple deprivation), and baseline score. ^3^BISS domain scores range from 1 to 9, where higher scores are more favourable.
