# Supplementary material for: Feasibility and acceptability of a brief routine weight management intervention for postnatal women embedded within the national child immunisation programme in primary care: randomised controlled cluster feasibility trial
Source: Trials. 2020 Sep 1;21:757. doi: 10.1186/s13063-020-04673-9 (PMC7466790; doi:10.1186/s13063-020-04673-9)
Supplement: Supplementary file 2 — Additional file 2. Weight control strategies. [file 13063_2020_4673_MOESM2_ESM.docx]

**Additional file 2.**

|  | **3-month follow-up** | | |
| --- | --- | --- | --- |
|  | **Intervention (N=15)^1^** | **Usual Care (N=12)** | **Adjusted mean difference^2^ (95% CI)** |
| Total WCSS Score^3^ Mean (SD, N) | 1.6 (0.6, 13) | 1.5 (0.6, 9) | -0.04 (-0.7, 0.6) |
| Minimum-Maximum | 0.8-2.7 | 0.5-2.2 |  |
| Missing | 2 | 3 |  |
| Dietary Choices^3^ Mean (SD, N) | 2.3 (0.7, 13) | 2.5 (0.8, 12) | -0.2 (-0.8, 0.3) |
| Minimum-Maximum | 1.2-3.7 | 0.6-3.4 |  |
| Missing | 2 | 0 |  |
| Self-monitoring strategies^3^ Mean (SD, N) | 0.9 (0.9, 14) | 0.6 (0.7, 12) | 0.4 (-0.7, 1.5) |
| Minimum-Maximum | 0-2.7 | 0-2.1 |  |
| Missing | 1 | 0 |  |
| Physical activity^3^ Mean (SD, N) | 1.4 (0.8, 14) | 1.2 (0.6, 11) | 0.2 (-0.5, 0.9) |
| Minimum-Maximum | 0-2.7 | 0.3-2.2 |  |
| Missing | 1 | 1 |  |
| Psychological coping^3^ Mean (SD, N) | 1.5 (0.5, 13) | 1.5 (0.7, 10) | -0.03 (-0.5, 0.4) |
| Minimum-Maximum | 0.7-2.7 | 0.6-2.6 |  |
| Missing | 2 | 2 |  |

^1^One intervention group participant withdrew prior to follow-up. ^2^Values >0 favour intervention. Adjusted for GP practice (random effect) and the two minimisation variables (GP size list and index of multiple deprivation). ^3^WCSS domain scores range from 0 to 4, where higher scores are more favourable.
