# Supplementary material for: Feasibility and acceptability of a brief routine weight management intervention for postnatal women embedded within the national child immunisation programme in primary care: randomised controlled cluster feasibility trial
Source: Trials. 2020 Sep 1;21:757. doi: 10.1186/s13063-020-04673-9 (PMC7466790; doi:10.1186/s13063-020-04673-9)
Supplement: Supplementary file 3 — Additional file 3. Perceptions of self-weighing. [file 13063_2020_4673_MOESM3_ESM.docx]

**Additional file 3.**

|  | **Weight Management**  **(N=15)^1,2^** |
| --- | --- |
| Over the past 3 months, how difficult was it to weigh yourself regularly? | |
| Mean (SD, N) | 5.1 (2.6, 13) |
| Minimum-Maximum | 1-8 |
| Missing | 2 |
| Over the past 3 months, how difficult was it to remember to weigh yourself regularly? | |
| Mean (SD, N) | 5.2 (2.6, 13) |
| Minimum-Maximum | 1-8 |
| Missing | 2 |
| Over the past 3 months, how helpful did you find regular self-weighing? | |
| Mean (SD, N) | 5.8 (2.3, 13) |
| Minimum-Maximum | 2-8 |
| Missing | 2 |
| Over the past 3 months, how frustrating was it to weigh yourself regularly? | |
| Mean (SD, N) | 5.2 (2.5, 13) |
| Minimum-Maximum | 1-8 |
| Missing | 2 |
| Over the past 3 months, how anxious did you feel because of weighing yourself regularly? | |
| Mean (SD, N) | 4.5 (2.6, 13) |
| Minimum-Maximum | 1-8 |
| Over the past 3 months, how self-conscious did you feel because of weighing yourself regularly? | |
| Mean (SD, N) | 4.2 (2.6, 13) |
| Minimum-Maximum | 1-8 |
| Missing | 2 |
| Over the past 3 months, I found weighing myself regularly to be positive/negative experience | |
| Mean (SD, N) | 5.7 (1.7, 13) |
| Minimum-Maximum | 4-8 |
| Missing | 2 |
| How likely are you to weigh yourself regularly after this study ends? | |
| Mean (SD, N) | 5.2 (2.8, 13) |
| Minimum-Maximum | 1-8 |
| Missing | 2 |

^1^One participant in the intervention group withdrew prior to follow-up. ^2^Scores range from 1 to 8, where higher scores are more favourable.
