# Supplementary material for: Feasibility and acceptability of a brief routine weight management intervention for postnatal women embedded within the national child immunisation programme in primary care: randomised controlled cluster feasibility trial
Source: Trials. 2020 Sep 1;21:757. doi: 10.1186/s13063-020-04673-9 (PMC7466790; doi:10.1186/s13063-020-04673-9)
Supplement: Supplementary file 4 — Additional file 4. Withdrawals, loss to follow-up and missing data. [file 13063_2020_4673_MOESM4_ESM.docx]

**Additional file 4.**

One participant allocated to the intervention group withdrew from the trial as they decided not to have their child immunised. One participant withdrew from the intervention but remained in the trial. All remaining participants (n=27) completed the follow-up visit for assessment of outcomes, although one participant allocated to the intervention group did not complete the follow-up questionnaires. Data regarding attendance at child immunisation appointments was not provided by practices for three participants (intervention: n=2; usual care: n=1). Weight record cards were returned blank (so are considered not returned) for three participants (intervention group).
